# Latent profiles of patients with borderline pathology based on the alternative DSM-5 model for personality disorders

**DOI:** 10.1186/s40479-021-00146-w

**Published:** 2021-02-11

**Authors:** Dominick Gamache, Claudia Savard, Philippe Leclerc, Maude Payant, Alexandre Côté, Jonathan Faucher, Mireille Lampron, Marc Tremblay

**Affiliations:** 1 Department of Psychology, Université du Québec à Trois-Rivières, C.P. 500, Trois-Rivières, QC G9A 5H7 Canada; 2 CERVO Brain Research Centre, Quebec City, QC Canada; 3 Interdisciplinary Research Centre on Intimate Relationship Problems and Sexual Abuse, Montreal, QC Canada; 4 Department of Educational Fundamentals and Practices, Université Laval, Quebec City, QC Canada; 5 Department of Psychology, Université du Québec à Montréal, Montreal, QC Canada; 6 School of Psychology, Université Laval, Quebec City, QC Canada; 7 CIUSSS-Capitale-Nationale, Quebec City, QC Canada

**Keywords:** DSM-5, Alternative model for personality disorders, Borderline personality disorder, Criterion A, Criterion B, Latent profile analysis

## Abstract

**Background:**

There have been multiple attempts to try to parse out heterogeneity within borderline pathology by identifying patient subtypes; thus far, these works have yielded few consistent results. Recent developments in the operationalization of borderline pathology may provide new opportunities to identify clinically and conceptually meaningful subgroups of patients. The Alternative DSM-5 Model for Personality Disorders (AMPD) offers a categorical-dimensional operationalization of Borderline personality disorder (BPD) that has yet to be tested for identification of patient subgroups. The purpose of the present study is to test whether the combination of the Criterion A elements (pertaining to level of severity) and the seven pathological facets from Criterion B that define BPD in the AMPD can yield meaningful patient profiles.

**Methods:**

A total of 211 outpatients from a specialized PD treatment program (133 women, *M*age = 33.66, *SD* = 10.97) were selected based on the presence of at least moderate borderline pathology according to cutoffs recently proposed for the Borderline Symptom List-23. Valid Criterion A (Self and Interpersonal Functioning Scale) and B (Personality Inventory for DSM-5 Faceted Brief Form) self-reports were administered to measure elements and facets that define BPD in the AMPD model; these variables were used as indicators in a latent profile analysis (LPA).

**Results:**

The optimal solution generated by LPA yielded four distinct profiles: (a) *Borderline traits*; (b) *Moderate pathology with Impulsivity*; (c) *Moderate pathology with Identity problems and Depressivity*; and (d) *Severe pathology*. Clinically meaningful distinctions emerged among profiles on AMPD indicators and external variables relevant to PD, especially aggression and impulsivity.

**Conclusions:**

Profiles reflected both the “severity” and “style” components imbedded within Criterion A and B of the AMPD, as they were mainly distinguished by a continuum of severity but also by some meaningful qualitative differences that may have important clinical implications for treatment planning and contracting. Results also suggest that the four Criterion A elements have independent value to identify important differences in patients with borderline pathology. They also highlight that some Criterion B facets that define BPD in the AMPD may be especially important to identify subgroups of patients, mainly Impulsivity and Depressivity.

**Supplementary Information:**

The online version contains supplementary material available at 10.1186/s40479-021-00146-w.

## Background

The definition of borderline pathology has remained somewhat elusive and contentious over the years [1]. Part of the difficulty in identifying the core feature(s) of “borderlineness” [2] may be attributable to its highly heterogeneous nature, which stems from conceptual, theoretical, and empirical issues. For instance, the polythetic approach to diagnose Borderline personality disorder (BPD) in the *Diagnostic and Statistical Manual of Mental Disorders* (DSM) [3] entails that up to 256 different permutations of its nine defining criteria can lead to a diagnosis. In various psychoanalytic-psychodynamic models (e.g. [4, 5],), borderline pathology holds a “double status” as both a distinct disorder and as a level of personality organization comprising many of the usual categorical disorders (e.g., narcissistic, antisocial), which may also contribute to the elusiveness of a consensual definition and operationalization of “borderlineness.”

Over the past years, multiple endeavors have focused on trying to parse out the heterogeneity within borderline pathology by identifying patient subtypes. These efforts can be categorized as either “variable-centered” or “person-centered” [6]. The former approach has typically focused on DSM BPD criteria, using exploratory (EFA) and confirmatory (CFA) factor-analytic strategies in order to reduce the number of criteria to a few core dimensions. Unfortunately, EFA studies have yielded unstable solutions that often pose conceptual challenges for interpretation, while CFA studies have failed to find definitive support for multidimensional models over a single-factor solution (see [6] for a summary). To further complicate matters, a factor-analytic study by Sharp et al. [7] that included a bifactor analysis found that the nine DSM BPD criteria only loaded on a general personality pathology factor, suggesting that BPD criteria may represent core features of general PD severity instead of a discrete disorder.

The “person-centered” approach, on the other hand, has used strategies such as latent class (or profile) analysis (LPA) or cluster analysis in order to identify meaningful subgroups of BPD patients. Most studies have focused on Section II DSM criteria (e.g. [8–10],), yielding subgroups mostly distinguishable based on a gradient of severity. Other studies have focused on indicators relevant for borderline pathology (e.g., affect experience/regulation, interpersonal patterns, PD comorbidity, levels of antisocial behavior and aggression, mistrustfulness [6, 11–15]), which yielded two to four profiles/clusters. While no consensus emerged regarding their composition, Smits et al. [14] outlined that most categorizations seem to feature subtype(s) with elements of internalizing (i.e., the propensity to experience depressed mood, distress, and fear) and externalizing (i.e., the propensity to experience disinhibitory symptoms) pathology [16]. Of note, a recent study using a combination of models (factor analysis, latent class analysis, factor mixture modeling) on a large sample of undergraduates (> 20,000) brought additional support for the importance of the internalizing-externalizing distinction. Specifically, analyzes yielded three subgroups, one asymptomatic along with two subtypes with subthreshold borderline symptomatology: an Unstable subtype with recklessness and self-damaging behaviors, and an Empty subtype with emptiness, dissociation, emotional distress, and attachment avoidance [17].

In sum, the heterogeneity within borderline pathology is far from resolved, and efforts aimed at parsing it have yielded few consistent results. However, the recent “paradigmatic shift” in the conceptualization of personality pathology (e.g. [18],), with the field decisively moving towards a dimensional framework, provides new opportunities to study this heterogeneity. One of the most influential models that emerged over the past few years is the Alternative Model for Personality Disorders (AMPD). It was introduced in Section III of the fifth edition of the DSM (DSM–5 [3]) in response to the well-documented shortcomings of the traditional, categorical model of personality disorders (e.g., see [19] for a review). The model includes two main components. Criterion A comprises four elements pertaining to impairments in one’s sense of self (Identity and Self-direction) and in interpersonal relationships (Empathy and Intimacy); it was conceived as a “severity” indicator [20]. Criterion B includes 25 maladaptive personality facets hierarchically organized into five broader domains, i.e., Negative Affectivity, Detachment, Antagonism, Disinhibition, and Psychoticism [21]; it was meant to be a “style” indicator. The model also retains six specific personality disorders which can be diagnosed based on “algorithms” represented by a combination of Criterion A elements (scores ≥2 on at least two Criterion A elements must be present to diagnose a personality disorder) and Criterion B facets (a polythetic approach similar to Section II PDs is retained, with the notable exception that for certain disorders, some facets must be present in order to diagnose a given PD); as such, the AMPD model might better be described as a “hybrid” rather than a purely dimensional model.

The BPD diagnosis was retained in the AMPD, along with Antisocial, Avoidant, Narcissistic, Obsessive-compulsive, and Schizotypal PD diagnoses. BPD diagnosis is given in presence of moderate or greater impairment in two or more Criterion A elements, and in presence of four or more of the following Criterion B facets (one of which must be e, f, or g): (a) Emotional lability; (b) Anxiousness; (c) Separation insecurity; (d) Depressivity; (e) Impulsivity; (f) Risk taking; and (g) Hostility. Research on the AMPD BPD diagnosis has shown adequate continuity with Section II BPD diagnosis (see [22] for a summary). It should be noted that the inclusion-exclusion of some Criterion B facets in the AMPD operationalization of BPD has been recently disputed. A meta-analytic study by Watters et al. [23] found that most Criterion B facets (17 out of 25) were significantly associated with AMPD BPD, while Risk Taking (despite being part of the AMPD BPD definition) was not. In another recent study, Mulay et al. [24] revealed that four facets (Anxiousness, Depressivity, Emotional lability, and Impulsivity) were identified by a group of expert clinicians as the key Criterion B indicators for borderline pathology, setting aside Hostility, Risk taking, and Separation insecurity as “weaker” indicators.

### The present study

The aim of the present study is to use the AMPD conceptualization in order to identify subtypes of patients with borderline pathology using LPA. Previous LPA research, mostly based on DSM Section II criteria, has yielded mixed results in BPD subtyping, i.e., generating latent profiles mainly distinguishable by a gradient of severity (see, e.g. [8],) rather than by qualitative differences. Therefore, the LPA approach for subtyping patients with borderline pathology should be considered as a stringent evaluation of the capacity of the AMPD to generate meaningful subgroups that would be qualitatively distinct from one another. By combining a gradient of severity (Criterion A) with a “stylistic” element (Criterion B), AMPD-based profiles might be able to generate qualitatively distinct profiles distributed along a severity continuum, which other approaches (e.g., based on DSM Section II criteria) could not readily achieve. As a secondary objective, the use of the four Criterion A elements and the seven Criterion B BPD facets as latent indicators should allow (a) contributing to the current debate [25–27] regarding the unidimensional versus multidimensional nature of Criterion A, by determining whether the four Criterion A elements are independently useful to uncover conceptually and clinically meaningful profiles of BPD patients; and (b) contributing to ongoing efforts [23, 24] aiming to identify which Criterion B facets are the most relevant in the description of BPD.

## Methods

### Participants and procedures

Participants were selected from a database of French-Canadian outpatients recruited during the intake procedure at a psychiatric outpatient clinic specialized for PD treatment in Quebec City, Canada. All were referred to the treatment center for an initial evaluation of suitability in the outpatient treatment program, following a reference by a psychiatrist or general physician for a suspected PD. Patients were first asked to complete a computerized self-report battery of questionnaires, and then took part in a clinical interview led by a clinical psychologist, who produced a detailed evaluation report. In line with the objectives of the present study, a total of 211 patients^1^ (133 women, *M*_age_ = 33.66, *SD* = 10.97) were selected based on the presence of at least moderate borderline pathology according to cutoffs recently proposed by Kleindienst et al. [28] for the short version of the Borderline Symptom List ( [29]; see below). According to these proposed grades of borderline symptom severity, 26.5% had a moderate level of pathology (score 0.7–1.7; *n* = 56), 40.8% had a high level (1.7–2.7; *n* = 86), 25.1% had a very high level (2.7–3.5; *n* = 53), and 6.6% had an extremely high level (3.5–4; *n* = 14). Almost all participants (97.2%) were of Caucasian-White ethnicity. Half (49.8%) were unemployed or on disability leave, while the others were full-time or part-time workers (34.6%), students (12.8%), or pensioners (2.8%). A majority (66.4%) were single, divorced, or widowed.

### Measures

#### Self-reported variables

The short version of the Borderline Symptom List (BSL-23 [29]; French validation by Nicastro et al. [30]) is a 23-item self-rating instrument assessing Borderline PD symptomatology. The instrument was used to guide participant selection, i.e., to identify prospective participants with at least moderate borderline pathology; it was also used as an external comparator among profiles. The BSL-23 covers DSM Section II BPD diagnostic criteria (e.g., affective instability, suicidality, transient psychotic symptoms) in addition to other affective experiences typical of borderline pathology (e.g., shame, self-criticism, mistrustfulness, and helplessness). The severity grades proposed by Kleindienst et al. [28] received robust empirical support from established assessments for psychopathology across three samples. Items are scored on a five-point Likert scale. The global score (MacDonald’s Omega [ω] = .91) was used in the present study.

The Self and Interpersonal Functioning Scale [31] is a 24-item self-report measure of the AMPD Criterion A. Items are rated on a five-point Likert scale (higher scores indicate higher dysfunction). It provides a global personality dysfunction score (ω = .80) and four subscale scores: Identity (ω = .60), Self-direction (ω = .68), Empathy (ω = .66), and Intimacy (ω = .68). Previous research on the SIFS using CFA yielded a second-order model, with four elements organized into a higher-order personality dysfunction factor [31]; meaningful patterns of associations with related psychological constructs were found for the four SIFS subscales. Content validity analysis of the SIFS items also showed promising results, and the severity level assessed by its items makes it very well suited to study populations with greater psychopathology [32]. In the present study, the four elements were used as LPA indicators, while the global score was used to contrast profiles.

The Personality Inventory for DSM-5 Faceted Brief Form (PID-5-FBF [33]; French validation by Roskam et al. [34]) is an abbreviated 100-item self-report version, based on item-response theory, of the original 220-item PID-5 [35]. It covers 25 pathological personality facets, hierarchically organized into five domains: Negative Affectivity (ω = .72), Detachment (ω = .84), Antagonism (ω = .91), Disinhibition (ω = .74), and Psychoticism (ω = .85). The official American Psychiatric Association scoring method (i.e., using only three facets per domain) was used to determine domain scores. Items are rated on a four-point Likert scale. In the present study, the seven facets (ω range = .74 [Depressivity] to .89 [Impulsivity]) that define BPD in the AMPD model were used as latent indicators, whereas the other 18 facets (ω range = .66 [Irresponsibility] to .91 [Attention-seeking]) and the five domains were used in subsequent analyses to describe and contrast profiles.

Along with a sociodemographic questionnaire, other instruments were used to assess symptoms commonly encountered in BPD, for profile characterization and comparison:

The 12-item short-form Buss-Perry Aggression Questionnaire (BPAQ-SF [36]; French validation by Genoud and Zimmerman [37]) covers four manifestations of aggression: Verbal (ω = .63), Physical (ω = .86), Anger (ω = .80), and Hostility (ω = .70). It also yields a global Trait Aggression score (ω = .86). Items are scored on a seven-point scale.

The 28-item Interpersonal Reactivity Index–French Version (IRI [38]; French validation by Gilet et al. [39]) measures empathy and its components. Two of its subscales were used in the present study: Perspective taking (the ability to adopt others’ point of view; ω = .83), which assesses the cognitive component, and Empathic concern (the motivation to care about others; ω = .80), which focuses on the affective component. Items are scored on a seven-point scale.

The Barratt Impulsiveness Scale (BIS-11 [40]; French validation by Baylé et al. [41]) is a 30-item questionnaire designed to assess three components of impulsiveness: Attentional (ω = .60), Motor (ω = .75), and Nonplanning (ω = .69). Items are scored on a four-point scale.

#### File-rated variables

Patient files were reviewed by two authors of the present study (DG, CS), to retrieve information pertaining to aggression, suicide attempts, and self-harm. Both raters have significant clinical experience with PD treatment (respectively 17 and 12 years). They both scored 20 randomly selected files for interrater agreement purposes (intra-class correlation [ICC] for aggression = 1.00; suicide attempts: ICC = .98, 95% CI [.94–.99]; self-harm: ICC = .84, 95% CI [.60–.94]). All remaining files were then scored by only one of the authors (DG). Most files (*n* = 168) included at least one detailed evaluation report with information pertaining to the three target clinical indicators. For each indicator, raters used a three-point scale (mid-points were allowed) to assess antecedents of aggression, suicide attempts, and self-harm: *no prior aggression/suicide attempts/self-harm* (0); *possible* (1), corresponding to rare or minor acts (i.e., one or two minor acts of violence or self-harm that did not cause/intend to cause serious injury or that did not result in hospitalization); or *confirmed* (2), corresponding to repeated or severe acting outs (i.e., at least three occurrences of minor acts, or one severe act causing/intending to cause injuries, death, or that resulted in hospitalization).

### Statistical analyses

A latent profile analysis was performed to determine the presence of distinct profiles of personality functioning using Mplus version 8.4^2^ [42]. Latent profiles were evaluated using the four SIFS elements (Identity, Self-direction, Empathy, and Intimacy) and the seven AMPD BPD facets (Anxiousness, Depressivity, Emotional lability, Hostility, Impulsivity, Risk taking, Separation insecurity) as parameters. After data standardization, latent models for six different class solutions were evaluated. Optimal class solution was determined based on model entropy (with a score between .8 and 1.0 indicating adequate classification precision), Akaike (AIC) and Bayesian (BIC) Information Criterion, Sample-Size Adjusted-BIC (SABIC), and Lo-Mendell-Rubin Adjusted Likelihood Ratio Test (LMRT). Lower values for the AIC, BIC, and SABIC metrics are indicative of a better-fitting model, while a significant difference on the LMRT between consecutive class solutions (i.e., *k* vs. *k* − 1) suggests that the *k* class solution has a better fit than the *k* − 1 solution [43]. Interpretability of the solution was also considered.

In a second step, latent profiles from the retained solution were contrasted on sociodemographic variables, comorbid AMPD personality disorders, and on external variables relevant to BPD, using Kruskal-Wallis tests for nonparametric mean comparisons as profiles were expected to be of different sizes and normality assumption was unlikely to be met for some variables. For contingency tables involving categorical data, Chi-square analyses were used.

## Results

### Latent profile analysis

Fit and quality indices from the six tested profile solutions are presented in Table 1; Supplementary Figure 1 displays the six tested solutions, with evolution of profile formation from one solution to the next. The four-profile solution was retained as the best fitting model; LMTR was the most decisive factor, as results showed that the four-profile solution fit the data significantly better than the three-profile solution, and that neither the five- nor the six-profile solution were an improvement over the preceding one. Entropy (.80) and interpretability for the four-profile solution were also adequate. Results from the AIC, BIC, and SABIC values were less definitive (AIC and SABIC decreased across all six profile solutions; BIC figure from the three-profile solution was the best, although by a very narrow margin).

**Table 1:** Latent Profile Analysis for Class Solutions 1 to 6 Using the Four Self and Interpersonal Functioning Scale Elements and the Seven Borderline Facets from the DSM-5 Alternative Model for Personality Disorders as Latent Profile Indicators

Classes (k)	LMRT	AIC	BIC	Sample-Size Adjusted BIC	Entropy
1	–	6328.252	6401.993	6332.283	–
2	264.706*	6083.425	6197.388	6089.655	.75
3	70.131	6036.201	6190.387	6044.630	.77
**4**	57.393**	**6001.915**	**6196.323**	**6012.543**	**.80**
5	38.713	5986.600	6221.230	5999.426	.83
6	41.678	5968.273	6243.125	5983.298	.81

*Note*. *LMRT* Lo-Mendell-Rubin Adjusted Likelihood Ratio Test, *AIC* Akaike Information Criteria, *BIC* Bayesian Information Criteria. Retained solution in **bold**

*p < .001. **p < .0001

**Fig. 1:**
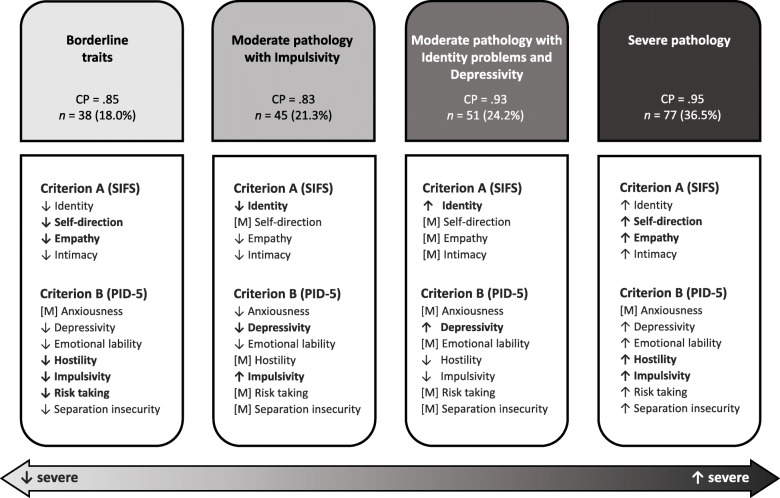
Final Four-Profile Solution, Using Latent Profile Analysis and Based on Criterion A Elements and Borderline Criterion B Facets from the Alternative Model for Personality Disorders. *Note.* CP = Classification probabilities. SIFS = Self and Interpersonal Functioning Scale. PID-5 = Personality Inventory for DSM-5 Faceted Brief Form. ↑ designates an element/facet whose profile mean score is above (> .25) the total sample mean. ↓ designates an element/facet whose profile mean score is below (< .25) the total sample mean. [M] designates an element/facet whose profile mean score is equivalent to the total sample mean (range − .25 to .25). **Elements/facets in boldface** designate elements/facets that represent core features of the corresponding profile

The four profiles were labeled: (a) *Borderline traits* (hereinafter labeled *Traits*); (b) *Moderate pathology with Impulsivity* (hereinafter labeled *Impulsive*); (c) *Moderate pathology with Identity problems and Depressivity* (hereinafter labeled *Identity/Depressivity*); and (d) *Severe pathology* (hereinafter labeled *Severe*; see Fig. 1 for a summary of the four profiles). Classification probabilities for the most likely latent profile membership were respectively .85, .83, .93, and .95. Participants from the *Traits* profile (*n* = 38; 18.0% of the sample) showed standardized scores below the total sample mean for all indicators but Anxiousness (*z*-score = .16), whereas PID-5 Impulsivity (− 1.50) and SIFS Self-direction (− 1.14) standardized scores were markedly low. The *Impulsive* profile (*n* = 45; 21.3%) was also characterized by *z*-scores below the total sample mean for almost all indicators, except for Impulsivity (.28). The *Identity/Depressivity* profile (*n* = 51; 24.2%) showed scores around the total sample mean for most indicators, with relatively higher elevations for SIFS Identity (.54) and PID-5 Depressivity (.37), and lower elevations for PID-5 Impulsivity (−.45) and Hostility (−.37). Finally, the *Severe* profile (*n* = 77; 36.5%) showed scores above the total sample mean on all indicators, the highest elevations being observed for PID-5 Impulsivity (.86), PID-5 Hostility (.74), SIFS Empathy (.74), and SIFS Self-direction (.73).

In a final step, the presence of an AMPD BPD diagnosis was determined using DSM-5 AMPD guidelines [3].^3^ No patient from the *Traits* profile had an AMPD BPD diagnosis; the proportion of patients from other profiles with the diagnosis were: *Impulsive*: *n* = 18 (40.0%); *Identity/Depressivity*: *n* = 23 (45.1%); and *Severe*: *n* = 70 (90.9%); there was a significant difference among profiles, χ^2^ = 91.51, *p* < .001, *d* = 1.75.

### Inter-profile differences on latent indicators

Supplementary Table 1 displays how the four profiles differ from each other on the 11 latent indicators.^4^ As profile subgroups were of different sizes, and some variables showed nonparametric distributions based on quantitative (e.g., Kolmogorov-Smirnov, Shapiro-Wilk) and visual indices (e.g., histogram), Kruskall-Wallis tests with two-tailed post-hoc comparisons using Bonferroni’s correction for multiple contrasts were selected to compare profiles. All indicators but PID-5 Anxiousness showed significant differences among profiles, with PID-5 Impulsivity appearing to be the most discriminant. There were relatively few differences between the *Traits* and the *Impulsive* profiles, the latter showing significantly higher figures on three indicators (SIFS Self-direction, PID-5 Impulsivity and Risk taking). More significant differences were observed between the *Traits* profile and the *Identity/Depressivity* (on six indicators) and the *Severe* (on 10 indicators) profiles, respectively. The *Impulsive* and the *Identity/Depressivity* profiles were significantly different on four indicators: SIFS Identity, PID-5 Depressivity and Emotional lability (higher scores for the *Identity/Depressivity* profile), and PID-5 Impulsivity (higher scores for the *Impulsive* profile). The *Severe* profile evidenced significantly higher scores in contrast with all three other profiles.

### Differences on criterion B domains and non-borderline facets

Supplementary Table 3 shows differences on the four profiles on PID-5 domains and facets not included in the BPD AMPD diagnosis. For most domains and facets, the *Traits* and the *Severe* profiles showed, respectively, the lowest and the highest indices of personality pathology. Negative Affectivity, Antagonism, and Disinhibition were the most discriminant domains. At the facet level, most differences were observed between the *Traits* and the *Severe* profiles, and there were some conceptually meaningful differences among the *Impulsive*, *Identity/Depressivity*, and *Severe* profiles (e.g., less Anhedonia in the *Impulsive* profile in contrast with the other two; more Callousness, Irresponsibility, and Suspiciousness in the *Severe* profile in contrast with the other two).

### Differences on Sociodemographic, diagnostic, and clinically-relevant variables

Table 2 displays additional meaningful distinctions among profiles. No sociodemographic variable was discriminant, while some comorbid AMPD diagnoses (Antisocial, Obsessive-compulsive, Schizotypal) showed a significant difference (with more comorbidity, as expected, in the *Severe* profile). The *Traits* profile, in contrast with *Severe*, showed fewer borderline traits (BSL-23), less severe personality pathology (SIFS), better Perspective taking (IRI), and less externalization (all five BPAQ-SF indicators, file-rated aggression, all three BIS-11 indicators). In contrast with the *Impulsive* profile, it showed fewer BPD traits (BSL-23) and less externalization (four of the BPAQ-SF indicators, BIS-11 Motor and Nonplanning). Compared with *Identity/Depressivity*, it showed fewer borderline traits (BSL-23), less severe personality pathology (SIFS), and less externalization (BPAQ-SF Anger and Hostility, BIS-11 Motor and Attentional). The *Impulsive* profile, in contrast with *Identity/Depressivity*, showed fewer borderline traits (BSL-23) and less severe personality pathology (SIFS). In comparison with *Severe*, it evidenced fewer borderline traits (BSL-23), less severe personality pathology (SIFS), better Perspective taking (IRI), and less externalization (four of the five BPAQ indicators, BIS-11 Attentional). The *Identity/Depressivity* profile, in contrast with the *Severe* one, showed less severe personality pathology (SIFS), better empathy (both IRI indicators), and less externalization (all five BPAQ-SF indicators, file-rated aggression, all three BIS-11 indicators).

**Table 2:** Between-Profile Comparisons on Sociodemographic, Diagnostic, and Clinical Variables (*N* = 211)

Sociodemographic, diagnostic, and clinical variables		*Borderline traits* (*n* = 38)	*Moderate pathology with Impulsivity* (*n* = 45)	*Moderate pathology with Identity problems and Depressivity* (*n* = 51)	*Severe pathology* (*n* = 77)	*H* or χ^2^	Post-hoc comparisons^a^ following a significant Kruskall-Wallis test, or Fisher Exact Test for χ^2^
Comorbid ASPD (AMPD)	Yes	0	1	0	9	13.30**	1 < 4*
	No	38	44	51	68		3 < 4*
Comorbid OCPD (AMPD)	Yes	0	2	2	10	12.70**	1 < 4*
	No	38	43	49	67		
Comorbid SZPD (AMPD)	Yes	2	4	5	20	8.67*	1 < 4*
	No	36	41	46	57		2 < 4*
							3 < 4*
BSL-23	*M* (Raw/*z*)	1.68/−.77	1.95/−.43	2.60/.40	2.58/.37	51.34***	1 < 3***,4***
	*SD* (Raw/*z*)	.54/.68	.77/.98	.56/.71	.79/1.00		2 < 3***,4***
	MR	57.84	79.53	131.63	128.26		
SIFS Global Score	*M* (Raw/*z*)	1.56/−1.06	1.74/−.69	2.17/.15	2.51/.83	124.21***	1 < 3***,4***
	*SD* (Raw/*z*)	.36/.72	.30/.59	.29/.58	.37/.73		2 < 3***,4***
	MR	42.26	59.50	116.57	157.63		3 < 4**
Number of BPD AMPD borderline facets	*M* (Raw/*z*)	2.34/−1.00	3.24/−.37	3.41/−.26	5.06/.88	108.81***	1 < 2**, 3**,4***
	*SD* (Raw/*z*)	.53/.37	1.11/.77	1.04/.72	1.12/.77		2 < 4***
	MR	43.20	84.80	92.76	158.15		3 < 4***
IRI Perspective-taking^b^	*M* (Raw/*z*)	5.21/.52	4.81/.23	4.70/.15	3.67/−.59	19.60***	1 > 4***
	*SD* (Raw/*z*)	.85/.61	.83/.60	1.36/.99	1.57/1.13		2 > 4*
	MR	70.85	58.95	57.11	36.10		3 > 4*
IRI Empathic concern^b^	*M* (Raw/*z*)	5.70/.22	5.21/−.21	5.88/.38	5.08/−.33	9.61*	3 > 4*
	*SD* (Raw/*z*)	.95/.85	1.08/.97	.90/.81	1.27/1.13		
	MR	59.83	46.17	65.36	44.04		
BPAQ-SF Physical	*M* (Raw/*z*)	1.58/−.68	2.83/.07	2.03/−.41	3.57/.57	52.87***	1 < 2**, 4***
	*SD* (Raw/*z*)	.85/.51	1.50/.89	1.28/.76	1.77/1.06		3 < 4***
	MR	61.93	113.76	81.45	139.67		
BPAQ-SF Verbal	*M* (Raw/*z*)	2.32/−.59	3.03/−.00	2.49/−.45	3.76/.59	49.42***	1 < 2*, 4***
	*SD* (Raw/*z*)	1.12/.91	.89/.72	1.03/.83	1.20/.97		2 < 4***
	MR	68.64	109.09	78.89	140.58		3 < 4***
BPAQ-SF Anger	*M* (Raw/*z*)	2.74/−.91	3.80/−.16	3.71/−.22	4.98/.68	72.44***	1 < 2*, 3*, 4***
	*SD* (Raw/*z*)	1.22/.87	1.33/.94	1.19/.84	.99/.70		2 < 4***
	MR	53.78	94.94	89.77	149.23		3 < 4***
BPAQ-SF Hostility	*M* (Raw/*z*)	3.32/−.64	3.51/−.48	4.10/−.03	4.96/.62	57.02***	1 < 3*, 4***
	*SD* (Raw/*z*)	1.28/.98	1.15/.88	1.28/.98	.92/.70		2 < 4***
	MR	68.49	74.59	103.86	144.29		3 < 4**
BPAQ-SF Trait	*M* (Raw/*z*)	2.49/−.91	3.29/−.17	3.08/−.36	4.34/.79	88.20***	1 < 2**, 4***
	*SD* (Raw/*z*)	.75/.69	.80/.74	.87/.80	.88/.81		2 < 4***
	MR	49.98	96.72	83.33	154.16		3 < 4***
BIS-11 Motor	*M* (Raw/*z*)	1.85/−1.05	2.35/−.11	2.31/−.19	2.79/.71	86.70***	1 < 2***, 3***, 4***
	*SD* (Raw/*z*)	.30/.56	.44/.83	.47/.87	.42/.79		2 < 4***
	MR	39.71	98.17	96.21	149.78		3 < 4***
BIS-11 Attentional	*M* (Raw/*z*)	2.26/−.77	2.49/−.31	2.63/−.01	2.92/.57	51.94***	1 < 3**, 4***
	*SD* (Raw/*z*)	.42/.86	.50/1.02	.42/.86	.40/.80		2 < 4***
	MR	60.93	85.13	104.80	141.23		3 < 4**
BIS-11 Nonplanning	*M* (Raw/*z*)	2.24/−.75	2.64/.04	2.53/−.17	2.85/.45	37.02***	1 < 2**, 4***
	*SD* (Raw/*z*)	.50/.98	.42/.82	.44/.87	.48/.95		3 < 4**
	MR	61.90	107.40	96.15	133.52		
File-rated aggression^c^	*M* (Raw/*z*)	.36/−.38	.85/.21	.41/−.32	.92/.30	16.92**	1 < 4**
	*SD* (Raw/*z*)	.66/.82	.93/1.15	.69/.85	.80/.98		3 < 4**
	MR	66.89	92.01	70.28	99.41		

*Note.* ^a^Two-tailed, using Bonferroni’s correction for multiple comparisons. ^b^Higher scores denote better functioning. For all other variables, higher scores denote more severe pathology. ^c^*n* = 166. *ASPD* Antisocial Personality disorder, *OCPD* Obsessive-compulsive Personality disorder, *SZPD* Schizotypal Personality disorder, *AMPD* Alternative DSM-5 Model for Personality Disorders, *BPD* Borderline personality disorder, *SIFS* Self and Interpersonal Functioning Scale, *BSL-23* 23-item Borderline Symptoms List, *IRI* Interpersonal Reactivity Index, *BPAQ-SF* 12-item version of the Buss-Perry Aggression Questionnaire, *BIS-11* Barratt Impulsiveness Scale, *MR* Mean rank. Only variables with statistically significant differences among profiles are shown

*p < .05. **p < .01. ***p < .001

## Discussion

The present study aimed to identify subtypes of patient with borderline pathology based on the recent operationalization of BPD proposed in the Alternative DSM-5 Model for Personality Disorders. The four Criterion A elements and the seven Criterion B facets that define BPD in the AMPD were used as indicators in a latent profile analysis. A four-profile solution was deemed optimal based on quantitative indices and interpretability.

The present findings support a hybrid dimensional-categorical approach to BPD diagnosis, in line with the conceptualization proposed in the AMPD model. Indeed, profiles were distributed along a continuum of severity (dimensional component), with meaningful qualitative differences between two profiles at an intermediate level of severity (categorical component). This is in contrast with previous research based on Section II DSM criteria [8–10] that found BPD subtypes only distributed along a continuum of severity. The two profiles with moderate severity included a similar proportion of patients who would qualify for a formal AMPD BPD diagnosis (*Impulsive* = 40%, *Identity/Depressivity* = 45.1%). Patients from the *Impulsive* subgroup had lower indices of borderline pathology, level of personality pathology, and negative affectivity; however, their file-rated aggression levels were on par with the most severe profile. On the other hand, the *Identity/Depressivity* subgroup showed indices of subjective distress and poorer mental health on AMPD indicators and external variables. The delineation between the two aforementioned profiles bears a resemblance to the externalizing—internalizing structure of psychopathology [16]. Previous studies reported that BPD symptoms lie at the intersection of the externalizing and internalizing-distress dimensions [45, 46]; the present findings support the usefulness of the externalizing-internalizing framework for borderline pathology, while also suggesting that for a sizeable proportion of patients, one proclivity might be more pronounced than the other (i.e., patients in the *Impulsive* profile did not show marked internalizing symptoms, while patients in the *Identity/Depressivity* group did not appear to show marked disinhibition). We must bear in mind, however, that the present research design was cross-sectional, and that longitudinal research would be necessary to determine whether patients remain in their profile over time or if they tend to “move” between the two profiles (e.g., they might be mainly distressed some days, and mainly disinhibited other days).

One of the key findings of the present study is that a few dimensions should be paid particular attention with patients with borderline features, especially at a moderate level of borderline pathology. While the data show a straight linear trend for most variables (from lowest to highest per increasing severity of the groups), there were a few notable exceptions, which appear to delineate two subgroups. Among the variables that define BPD in the AMPD, Criterion A Identity, along with Criterion B Impulsivity and Risk Taking, appear to be key differentiating variables; a few other indicators (PID-5 Submissiveness, Empathic Concern, and physical aggression) also showed non-linear relations across profiles. These considerations may be especially important for assessment and treatment contracting, as previous studies suggest that the aforementioned profiles, and the variables that define them, may be associated with different treatment courses and outcomes. Impulsivity [47, 48] and aggression (e.g. [49],), which were more prominent in the *Impulsive* profile, have been identified as dropout predictors in previous research with PD patients; this might suggest, at first glance, that the *Impulsive* group has a poorer treatment prognosis. Furthermore, in a recent study conducted in a large sample of psychiatric patients, AMPD Disinhibition (which includes the Impulsivity and Risk taking facets) was associated with a twofold increase in dropout risk [50]. It should be noted that another study found no role for Disinhibition in the prediction of dropout for patients with Cluster C and mild Cluster B PDs [51].

The present study has two other noteworthy contributions. First, results support the claim that the four Criterion A elements have a value on their own. Indeed, SIFS Identity, Self-direction, and Empathy all played a significant role in differentiating among profiles. The central role of identity-related impairment to borderline pathology, emphasized in influential theoretical and clinical models [52–54], was also supported by the present findings as Identity was a central factor in discriminating between the two profiles with moderate severity. Second, the present results also add to the ongoing discussion regarding the optimal combination of Criterion B facets that are the most descriptive of borderline pathology. The PID-5 facets of Impulsivity and Depressivity were the most useful, as shown by their ability to discriminate between the two intermediate profiles; this is in line with experts’ judgement about which facets are the most important for BPD description in the AMPD operationalization [24]. Of note, Anxiousness did not contribute at all to the distinction among profiles, despite being identified by PD scholars as one of the four most important BPD facets in the AMPD formulation [24]. A “ceiling effect” might be in cause here, as all profiles showed very high elevations (range 2.13–2.49 out of 3.0) for this facet. This might suggest that Anxiousness has poor discriminant value in samples where severity of borderline pathology is moderate or greater, and that its discriminant value is more obvious between BPD and other AMPD diagnoses, and not “within” BPD. While we believe that testing the actual AMPD model was the adequate initial step, future research should also investigate whether a more empirically-driven set of facets (e.g., identified through meta-analytic data [23] and expert ratings [24]) might offer a better representation of borderline pathology.

The most critical limitation to the present findings is the absence of replication using an independent clinical sample. The robustness and generalizability of the profile solution is unknown, and we cannot rule out that the resulting subgroups may be limited to this specific sample. Latent profile analyses often do not replicate from one study to another, or only partially do so, which can be an indication of poor replicability or could reflect methodological differences (e.g., different latent indicators; different sample composition)—or both; furthermore, despite some commendable exceptions [55], very few studies offer replication of their profile solutions, especially with clinical samples.

Two factors might partially mitigate concerns regarding the replicability of the present findings. First, our profile solution aligns with established theoretical-empirical frameworks of BPD and psychopathology. The extensively studied externalizing-internalizing dimension [16, 45, 46] appears to be central in distinguishing between two intermediate subgroups in terms of severity and dysfunction, one with higher externalizing features, the other with higher internalizing features. In the recently developed Hierarchical Taxonomy of Psychopathology (HiTOP [56]), a dimensional nosology of mental disorders based on their observed covariation across empirical studies, BPD is one of the very few diagnoses that would appear to belong to both an “internalizing” (distress) and an “externalizing” (antagonistic) spectra, which is also in line with our profile solution. The externalizing-internalizing dimension was also a key factor in a number of previous studies of BPD subtypes [14]. Second, our results partially replicate those reported by Clark et al. [55] in the only study thus far, to our best knowledge, that used the AMPD framework to identify subgroups of outpatients (although not specifically borderline). Clark et al. found evidence for three groups: one with primarily interpersonal problems, one with self-pathology and emotional dysregulation/negative affectivity, and one with a more severe presentation showing both types of problems. While there are evident resemblances between our *Identity/Depressivity* profile and Clark et al.’s self-pathology/negative affectivity profile, and between the severe profiles found in both studies, there are also divergences between the two classifications, as impulsivity (in our study) and interpersonal pathology (in Clark et al.’s) appeared to have inconsistent value across studies to assign patients to profiles. Our results also echo findings from Johnson and Levy [17] who obtained, using Finite mixture modeling, an Unstable (with externalizing problems) and an Empty (with internalizing problems) subtype of subthreshold BPD in an undergraduate sample. Of note, while the authors from the aforementioned study did not retain a four-profile solution yielded by latent class analysis as their optimal solution, it showed remarkable parallels with our own findings (their profiles were labeled Asymptomatic, Affective/impulsive, Empty/identity disturbed, and Highly symptomatic “BPD”). However, given that the present results only partially replicate these previous findings [17, 55], the issue remains a valid concern that can only be resolved through further replication.

This study has other notable limitations. There were methodological choices that might limit the value of some findings. Although the present study was based on a dimensional framework, some categorical decisions had to be made (an irony which is not lost on Clark et al. [55]); this required selection of thresholds which, in retrospective, might not have been optimal. The most glaring example of this is the so-called “rational method” of ≥2 to determine the presence of a Criterion B facet (e.g. [44],). Using this threshold led to a somewhat surprising “floor effect” for a number of facets; this resulted in very low prevalence estimates for some other AMPD diagnoses. Using a “rounded” approach for scores of 1.5 and above, which was implemented in other studies (e.g. [24],), might be indicated in future studies. The underwhelming comparisons among profiles for AMPD comorbidity should therefore be interpreted with this caveat in mind. Also, the external validity of conclusions is obviously contingent upon the extraction of the optimal number of profiles; it must be acknowledged that our retained four-profile solution was not completely unambiguous (as shown by other plausible alternatives; see Table 1 and Supplementary Figure 1). Data were mostly collected through self-report questionnaires, which comes with the risk of dishonesty and/or poor insight in some cases; however, evidence supporting the validity and usefulness of self-ratings of personality pathology is accumulating [57], as most patients seem to report considerable levels of personality pathology through self-report assessment [58]. While file-rated aggression showed meaningful distinctions among profiles, the absence of significant results for file-rated suicidality and self-harm might point to a lack of sensitivity of the scales designed to rate them, to a lack of power in contrast with other indicators and variables (as there were a number of missing data), or to an “authentic”—albeit surprising—absence of difference among profiles on these critical outcome variables. In addition, replications should include more participants from diverse ethnic backgrounds as cultural factors play a role in BPD diagnosis and prevalence [59].

Ultimately, the defining test of the validity of the profiles identified in the present analysis will rely on their clinical usefulness, i.e., whether they are clinically helpful for treatment planning and contracting; whether they provide relevant information on treatment trajectories, including response to treatment and dropout risk; and whether some treatment approaches might be better suited for patient from one profile or another [14]. Clark et al. [55] have suggested that AMPD-based profiles can be useful for differential treatment planning based on transdiagnostic treatment targets (e.g., emotional dysregulation, interpersonal issues), a suggestion with which we concur.

Another central issue in determining the usefulness of the present findings pertains to their incremental validity over and beyond a linear combination of the AMPD indicator variables. In further replications, a meaningful examination of the incremental validity of the present profiles will require the inclusion of a broader range of variables to cover a wide range of symptoms (e.g., internalizing, thought problems) and outcomes (e.g., behavioral, clinical, occupational, relational) relevant for BPD.

## Conclusion

This study used a “person-centered” approach to tackle the issue of heterogeneity in patients with borderline pathology, using latent profile analysis to generate subtypes based on the emerging Alternative DSM-5 Model for Personality Disorders operationalization of BPD. Four profiles were identified, which reflected both the “severity” and “style” components imbedded within Criterion A and B of the AMPD. They were mainly distinguished by a continuum of severity but also by some meaningful qualitative differences, especially at an intermediate level of severity; these differences likely have important clinical implications, notably for treatment planning and contracting. Results also contribute to the healthy ongoing discussions regarding the optimal conceptualization of Criterion A, suggesting that its four constituent elements have independent value and that they can be useful to identify important differences in patients with borderline pathology. They also support the centrality of identity impairment in the conceptualization of borderline pathology, as it was a key element for profile formation. The present findings also highlight that some Criterion B facets that define Borderline PD in the AMPD may be especially important to identify subgroups of patients, mainly Impulsivity and Depressivity. Replication and demonstration of the clinical utility of the profiles identified in the current study (e.g., for treatment planning-contracting, for tailoring treatment to key pathological dimensions, and for prediction of treatment course and outcome) will be of utmost importance in determining the value of the present findings.

## Supplementary Information

**Additional file 1: Table S1**. Between-Group Comparisons on the 11 Latent Indicators from the Self and Interpersonal Functioning Scale and the Personality Inventory for DSM-5 Faceted Brief Form (*N* = 211). **Table S2**. Bivariate Pearson Correlations between Latent Profile Indicators from the Self and Interpersonal Functioning Scale and the Personality Inventory for DSM-5 Faceted Brief Form (*N* = 211). **Table S3**. Between-Profile Comparisons on the Personality Inventory for DSM-5 Domains and Non-Borderline Facets (*N* = 211). **Figure S1**. Conceptual Diagram of the Series of Latent Class Analyses (One to Six) with Tentative Designation for All Profiles Extracted during Each Step.

## Data Availability

The datasets generated and/or analyzed during the current study are not publicly available based on the terms on which the study was approved by the ethics committee but are available from the corresponding author on reasonable request.

## Abbreviations

AIC: Akaike Information Criterion
AMPD: Alternative DSM-5 Model for Personality Disorders
BIC: Bayesian Information Criterion
BIS-11: Barratt Impulsiveness Scale
BPAQ-SF: Buss-Perry Aggression Questionnaire Short Form
BPD: Borderline personality disorder
BSL-23: Borderline Symptom List-23
CFA: Confirmatory factor analysis
CI: Confidence interval
DSM: *Diagnostic and Statistical Manual of Mental Disorders*
EFA: Exploratory factor analysis
HiTOP: Hierarchical Taxonomy of Pathology
ICC: Intra-class correlation
IRI: Interpersonal Reactivity Index
LMRT: Lo-Mendell-Rubin Adjusted Likelihood Ratio Test
LPA: Latent profile analysis
PID-5-FBF: Personality Inventory for DSM-5 Faceted Brief Form
PD: Personality disorder(s)
SABIC: Sample-Size Adjusted Bayesian Information Criterion
SIFS: Self and Interpersonal Functioning Scale

## Footnotes

1 An additional participant was excluded from further analyses for clearly discernable underreporting.

2 All other analyses were executed with SPSS 26.0, with the exception of ω computation, which was done with JASP 0.13.1.

3 For Criterion A, the presence of two or more elements is required; we used SIFS proposed clinical cut-off scores (based on Receiver Operating Characteristic curves) for the four elements [31] to rule on the presence/absence of an element. For Criterion B, we used the DSM-5 AMPD threshold for BPD facets (i.e., presence of four or more facets that must include either Impulsivity, Risk taking, or Hostility; a facet was considered present using a threshold of ≥2, in line with the “rational method” described in the PID-5 literature, e.g. [44],).

4 Supplementary Table 2 provides zero-order bivariate Pearson correlations among all 11 latent indicators.
