# Supplementary material for: Latent profiles of patients with borderline pathology based on the alternative DSM-5 model for personality disorders
Source: Borderline Personal Disord Emot Dysregul. 2021 Feb 11;8:4. doi: 10.1186/s40479-021-00146-w (PMC7876791; doi:10.1186/s40479-021-00146-w)
Supplement: Supplementary file 1 — Additional file 1: Table S1. Between-Group Comparisons on the 11 Latent Indicators from the Self and Interpersonal Functioning Scale and the Personality Inventory for DSM-5 Faceted Brief Form (N = 211). Table S2. Bivariate Pearson Correlations between Latent Profile Indicators from the Self and Interpersonal Functioning Scale and the Personality Inventory for DSM-5 Faceted Brief Form (N = 211). Table S3. Between-Profile Comparisons on the Personality Inventory for DSM-5 Domains and Non-Borderline Facets (N = 211). Figure S1. Conceptual Diagram of the Series of Latent Class Analyses (One to Six) with Tentative Designation for All Profiles Extracted during Each Step. [file 40479_2021_146_MOESM1_ESM.docx]

**Supplementary Table 1**

*Between-Group Comparisons on the 11 Latent Indicators from the Self and Interpersonal Functioning Scale and the Personality Inventory for DSM-5 Faceted Brief Form (N = 211)*

| Latent profile indicators | |  | *Borderline traits*  (*n* = 38) | *Moderate pathology with Impulsivity* (*n* = 45) | *Moderate pathology with Identity problems and Depressivity*  (*n* = 51) | *Severe pathology*  (*n* = 77) | *H* | Post-hoc comparisons^a^ following a significant Kruskall-Wallis test |
| --- | --- | --- | --- | --- | --- | --- | --- | --- |
| SIFS Identity | *M* (Raw/*z*)  *SD* (Raw/*z*)  MR | | 2.41/-.50  .50/.78  70.59 | 2.15/-.90  .51/.80  49.06 | 3.08/.54  .48/.61  139.42 | 3.03/.47  .50/.78  134.62 | 84.62*** | 1 < 3***, 4***  2 < 3***, 4*** |
| SIFS Self-direction | *M* (Raw/*z*)  *SD* (Raw/*z*)  MR | | 1.20/-1.14  .52/.62  36.17 | 1.94/-.25  .57/.68  88.59 | 2.12/-.04  .58/.69  104.87 | 2.76/.73  .69/.83  151.38 | 96.44*** | 1 < 2**, 3***, 4***  2 < 4***  3 < 4*** |
| SIFS Empathy | *M* (Raw/*z*)  *SD* (Raw/*z*)  MR | | .82/-.83  .53/.68  58.22 | 1.14/-.43  .57/.73  80.20 | 1.40/-.09  .60/.77  101.95 | 2.05/.74  .72/.91  149.81 | 76.64*** | 1 < 3**, 4***  2 < 4***  3 < 4*** |
| SIFS Intimacy | *M* (Raw/*z*)  *SD* (Raw/*z*)  MR | | 1.67/-.28  .87/1.01  87.30 | 1.59/-.37  .76/.87  83.27 | 1.89/-.03  .75/.86  104.33 | 2.26/.40  .87/1.00  129.62 | 21.45*** | 1 < 4**  2 < 4*** |
| PID-5 Anxiousness | *M* (Raw/*z*)  *SD* (Raw/*z*) | | 2.49/.16  .59/.89 | 2.13/-.39  .79/1.20 | 2.44/.08  .62/.95 | 2.43/.07  .61/.93 | 8.33* | n.s. |
| PID-5 Depressivity | *M* (Raw/*z*)  *SD* (Raw/*z*)  MR | | 1.49/-.39  .71/.96  81.47 | 1.26/-.70  .66/.89  62.23 | 2.06/.37  .55/.74  128.53 | 2.09/.42  .66/.89  128.76 | 47.55*** | 1 < 3**, 4**  2 < 3***, 4*** |
|  |  | |  |  |  |  |  |  |
| PID-5 Emotional  lability | *M* (Raw/*z*)  *SD* (Raw/*z*)  MR | | 1.88/-.43  .74/1.09  79.83 | 1.85/-.46  .71/1.04  75.56 | 2.25/.12  .62/.92  111.58 | 2.47/.45  .47/.69  133.11 | 34.52*** | 1 < 4***  2 < 3*, 4*** |
|  |  | |  |  |  |  |  |  |
| PID-5 Hostility | *M* (Raw/*z*)  *SD* (Raw/*z*)  MR | | 1.01/-.75  .68/.87  61.95 | 1.49/-.14  .64/.82  95.58 | 1.30/-.37  .60/.76  80.76 | 2.18/.74  .62/.80  150.99 | 73.16*** | 1 < 4***  2 < 4***  3 < 4*** |
|  |  | |  |  |  |  |  |  |
| PID-5 Impulsivity | *M* (Raw/*z*)  *SD* (Raw/*z*)  MR | | .42/-1.50  .38/.45  22.42 | 1.91/.28  .47/.56  120.76 | 1.30/-.45  .38/.46  71.13 | 2.40/.86  .41/.49  161.72 | 156.13*** | 1 < 2***, 3**, 4***  2 > 3***, < 4**  3 < 4*** |
|  |  | |  |  |  |  |  |  |
| PID-5 Risk taking | *M* (Raw/*z*)  *SD* (Raw/*z*)  MR | | .63/-.85  .80/.89  55.26 | 1.48/.11  .89/.99  112.77 | 1.22/-.18  .85/.95  95.49 | 1.78/.44  .70/.78  134.05 | 44.90*** | 1 < 2***, 3*, 4***  3 < 4** |
|  |  | |  |  |  |  |  |  |
| PID-5 Separation insecurity | *M* (Raw/*z*)  *SD* (Raw/*z*)  MR | | 1.39/-.40  .83/.98  81.03 | 1.52/-.25  .81/.94  89.32 | 1.86/.16  .71/.83  113.28 | 1.95/.26  .89/1.05  123.25 | 16.73** | 1 < 4**  2 < 4* |

*Note*. ^a^ Two-tailed, using Bonferroni's correction for multiple comparisons. SIFS = Self and Interpersonal Functioning Scale; MR = Mean Rank; ID = Identity; SD = Self-direction; EMP = Empathy; INT = Intimacy; PID-5 = Personality Inventory for DSM-5 Faceted Brief Form. Higher scores indicate more severe dysfunction. Mean rank not shown in the absence of significant post-hoc comparisons.

* *p* < .05. ** *p* < .01. *** *p* < .001.

**Supplementary Table 2**

*Bivariate Pearson Correlations between Latent Profile Indicators from the Self and Interpersonal Functioning Scale and the Personality Inventory for DSM-5 Faceted Brief Form* *(N = 211)*

|  | SIFS  ID | SIFS  SD | SIFS  EMP | SIFS  INT | PID-5 ANX | PID-5 DEP | PID-5 EML | PID-5 HOS | PID-5 IMP | PID-5  RT | PID-5 SEP |
| --- | --- | --- | --- | --- | --- | --- | --- | --- | --- | --- | --- |
| SIFS ID |  | .40*** | .25*** | .22** | .11 | .42*** | .22** | .10 | .15* | .06 | .18** |
| SIFS SD |  |  | .46*** | .27*** | -.06 | .14* | .21** | .32*** | .61*** | .29*** | .22** |
| SIFS EMP |  |  |  | .41*** | .04 | .19** | .15* | .40*** | .40*** | .23** | .20** |
| SIFS INT |  |  |  |  | -.03 | .24** | -.01 | .15* | .15* | .16* | -.07 |
| PID-5 ANX |  |  |  |  |  | .16* | .20** | .13 | -.07 | -.26*** | .13 |
| PID-5 DEP |  |  |  |  |  |  | .13 | .08 | .11 | .12 | .08 |
| PID-5 EML |  |  |  |  |  |  |  | .37*** | .22** | .03 | .22** |
| PID-5 HOS |  |  |  |  |  |  |  |  | .44*** | .10 | .07 |
| PID-5 IMP |  |  |  |  |  |  |  |  |  | .44*** | .19** |
| PID-5 RT |  |  |  |  |  |  |  |  |  |  | .01 |

Note. SIFS = Self and Interpersonal Functioning Scale; ID = Identity; SD = Self-direction; EMP = Empathy; INT = Intimacy; PID-5 = Personality Inventory for DSM-5 Faceted Brief Form; ANX = Anxiousness; DEP = Depressivity; EML = Emotional lability; HOS = Hostility; IMP = Impulsivity; RT = Risk taking; SEP = Separation insecurity.

* *p* < .05. ** *p* < .01. *** *p* < .001.

**Supplementary Table 3**

*Between-Profile Comparisons on the Personality Inventory for DSM-5 Domains and Non-Borderline Facets (N = 211)*

| PID-5 Domains and non-borderline facets |  | | *Borderline traits*  (*n* = 38) | *Moderate pathology with Impulsivity*  (*n* = 45) | *Moderate pathology with Identity problems and Depressivity*  (*n* = 51) | *Severe pathology*  (*n* = 77) | *H* | Post-hoc comparisons^a^ following a significant Kruskall-Wallis test |
| --- | --- | --- | --- | --- | --- | --- | --- | --- |
| **Domains** |  | |  |  |  |  |  |  |
|  |  | |  |  |  |  |  |  |
| Negative Affectivity | | *M* (Raw/*z*)  *SD* (Raw/*z*)  MR | 1.92/-.36  .47/.96  81.16 | 1.83/-.54  .47/.97  72.34 | 2.18/.17  .45/.92  115.68 | 2.28/.38  .44/.90  131.37 | 34.50*** | 1 < 4***  2 < 3**, 4*** |
|  | |  |  |  |  |  |  |  |
| Detachment | | *M* (Raw/*z*)  *SD* (Raw/*z*) | 1.44/-.21  .63/1.06 | 1.39/-.29  .61/1.02 | 1.62/.10  .50/.85 | 1.68/.20  .60/1.01 | 8.69* | n.s. |
| Antagonism | | *M* (Raw/*z*)  *SD* (Raw/*z*)  MR | .32/-.64  .30/.47  65.67 | .70/-.05  .61/.94  104.78 | .58/-.24  .54/.84  92.35 | 1.07/.51  .70/1.09  135.66 | 37.44*** | 1 < 2*, 4***  2 < 4*  3 < 4*** |
|  | |  |  |  |  |  |  |  |
| Disinhibition | | *M* (Raw/*z*)  *SD* (Raw/*z*)  MR | .92/-1.26  .46/.80  34.72 | 1.69/.08  .39/.68  108.99 | 1.49/-.27  .39/.69  84.31 | 2.07/.75  .39/.67  153.79 | 105.79*** | 1 < 2***, 3**, 4***  2 < 4***  3 < 4*** |
|  | |  |  |  |  |  |  |  |
| Psychoticism | | *M* (Raw/*z*)  *SD* (Raw/*z*)  MR | .67/-.50  .49/.81  78.97 | .94/-.04  .72/1.19  100.21 | .92/-.08  .51/.85  103.12 | 1.17/.33  .58/.95  127.10 | 20.22*** | 1 < 4*** |
| **Non-borderline facets** | |  |  |  |  |  |  |  |
| Anhedonia | | *M* (Raw/*z*)  *SD*  MR | 1.75/-.35  .72/1.03  85.04 | 1.66/-.48  .77/.78  79.01 | 2.12/.17  .54/1.11  114.02 | 2.24/.34  .63/.90  126.80 | 23.36*** | 1 < 4**  2 < 3*, 4*** |
|  | |  |  |  |  |  |  |  |
| Attention-Seeking | | *M* (Raw/*z*)  *SD* (Raw/*z*)  MR | .85/-.56  .85/.87  71.78 | 1.33/-.06  .96/.98  102.98 | 1.32/-.06  .84/.86  102.53 | 1.73/.36  1.00/1.03  126.95 | 21.45*** | 1 < 4*** |
| Callousness | | *M* (Raw/*z*)  *SD* (Raw/*z*)  MR | .26/-.52  .53/.74  69.71 | .56/-.10  .68/.95  100.68 | .37/-.37  .49/.69  83.94 | 1.04/.56  .75/1.05  141.63 | 48.85*** | 1 < 4***  2 < 4**  3 < 4*** |
|  | |  |  |  |  |  |  |  |
| Cog./percept.dysregulation | | *M* (Raw/*z*)  *SD* (Raw/*z*)  MR | .33/-.39  .47/.73  81.89 | .61/.06  .70/1.10  106.46 | .57/-.00  .54/.84  111.36 | .68/.16  .71/1.11  114.08 | 8.12* | 1 < 4* |
|  | |  |  |  |  |  |  |  |
| Deceitfulness | | *M* (Raw/*z*)  *SD* (Raw/*z*)  MR | .45/-.64  .45/.47  64.90 | .89/-.03  .74/.96  105.99 | .78/-.24  .74/.80  94.05 | 1.29/.49  .91/1.09  134.65 | 37.87*** | 1 < 2**, 4***  3 < 4*** |
|  | |  |  |  |  |  |  |  |
| Distractibility | | *M* (Raw/*z*)  *SD* (Raw/*z*)  MR | 1.71/-.50  .89/1.14  85.21 | 2.11/.00  .61/.78  109.39 | 2.12/.02  .82/1.05  98.89 | 2.29/.24  .73/.93  118.99 | 13.76** | 1 < 4* |
|  | |  |  |  |  |  |  |  |
| Eccentricity | | *M* (Raw/*z*)  *SD* (Raw/*z*)  MR | 1.13/-.46  .76/.91  78.59 | 1.32/-.23  .96/1.16  92.88 | 1.48/-.04  .80/.96  103.20 | 1.95/.38  .89/.83  129.05 | 21.00*** | 1 < 4***  2 < 4** |
|  | |  |  |  |  |  |  |  |
| Grandiosity | | *M* (Raw/*z*)  *SD* (Raw/*z*)  MR | .24/-.39  .38/.59  87.84 | .46/-.05  .68/1.05  102.37 | .36/-.20  .50/.76  95.83 | .73/.36  .76/1.16  123.82 | 12.76** | 1 < 4***  3 < 4* |
|  | |  |  |  |  |  |  |  |
| Intimacy avoidance | | *M* (Raw/*z*)  *SD* (Raw/*z*) | 1.11/-.06  .93/1.03 | 1.13/-.03  .89/.99 | 1.24/.08  .88/.98 | 1.15/-.01  .92/1.02 | 0.67 | n.s. |
| Irresponsi-bility | | *M* (Raw/*z*)  *SD* (Raw/*z*)  MR | .63/-.69  .60/.80  64.74 | 1.04/-.14  .62/.83  99.23 | 1.04/-.14  .69/.92  97.93 | 1.52/.51  .74/.99  135.86 | 37.96*** | 1 < 4***  2 < 4**  3 < 4** |
|  | |  |  |  |  |  |  |  |
| Manipula-tiveness | | *M* (Raw/*z*)  *SD* (Raw/*z*)  MR | .45/-.58  .45/.55  70.74 | .89/-.05  .74/.90  105.90 | .79/-.18  .74/.90  96.32 | 1.29/.43  .91/1.12  129.87 | 26.20*** | 1 < 4***  3 < 4* |
|  | |  |  |  |  |  |  |  |
| Perseveration | | *M* (Raw/*z*)  *SD* (Raw/*z*)  MR | 1.34/-.54  .65/.93  72.55 | 1.49/-.31  .65/.93  85.89 | 1.84/.18  .63/.90  116.41 | 1.94/.33  .69/.99  127.36 | 27.53*** | 1 < 3**, 4***  2 < 4** |
|  | |  |  |  |  |  |  |  |
| Restricted affectivity | | *M* (Raw/*z*)  *SD* (Raw/*z*)  MR | .82/-.35  .75/.99  85.21 | 1.11/.03  .69/.91  109.39 | 1.00/-.12  .68/.89  98.89 | 1.26/.23  .81/1.07  118.99 | 8.82* | 1 < 4* |
|  | |  |  |  |  |  |  |  |
| Rigid perfectionism | | *M* (Raw/*z*)  *SD* (Raw/*z*) | 1.79/.03  .78/.97 | 1.56/-.26  .76/.94 | 1.80/.05  .80/.99 | 1.85/.10  .85/1.05 | 4.83 | n.s. |
| Submissi-veness | | *M* (Raw/*z*)  *SD* (Raw/*z*)  MR | 1.43/-.08  .73/.94  101.43 | 1.25/-.30  .74/.95  89.59 | 1.90/.53  .74/.95  136.32 | 1.38/-.13  .76/.97  97.76 | 17.66** | 1 < 3*  2 < 3***  3 > 4** |
|  | |  |  |  |  |  |  |  |
| Suspicious-ness | | *M* (Raw/*z*)  *SD* (Raw/*z*)  MR | 1.15/-.31  .61/.83  86.67 | 1.18/-.28  .75/1.02  89.47 | 1.25/-.18  .64/.87  95.98 | 1.71/.44  .73/1.00  132.14 | 23.08*** | 1 < 4**  2 < 4**  3 < 4** |
|  | |  |  |  |  |  |  |  |
| Unusual beliefs and experiences | | *M* (Raw/*z*)  *SD* (Raw/*z*)  MR | .55/-.39  .64/.87  80.66 | .91/.11  .78/.57  111.49 | .72/-.15  .58/.80  100.17 | 1.00/.23  .78/1.07  119.16 | 11.19* | 1 < 4** |
|  | |  |  |  |  |  |  |  |
| Withdrawal | | *M* (Raw/*z*)  *SD* (Raw/*z*) | 1.45/-.09  .78/1.05 | 1.37/-.21  .79/1.06 | 1.51/-.01  .63/.85 | 1.65/.17  .76/1.02 | 4.41 | n.s. |

*Note*. ^a^ Two-tailed, using Bonferroni's correction for multiple comparisons. PID-5 = Personality Inventory for DSM-5 Faceted Brief Form; MR = Mean rank; Cog./percept.dysregulation = Cognitive and perceptual dysregulation. Higher scores indicate more severe dysfunction. Mean rank not shown in the absence of significant post-hoc comparisons.

* *p* < .05. ** *p* < .01. *** *p* < .001.

**Supplementary Figure 1**

*Conceptual Diagram of the Series of Latent Class Analyses (One to Six) with Tentative Designation for All Profiles Extracted during Each Step*

*
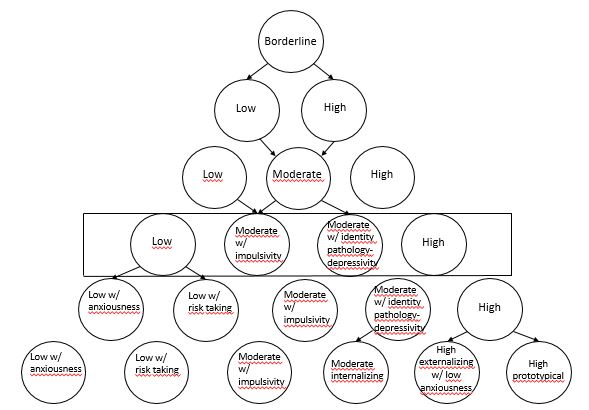
*

*Note.* Arrows were used to illustrate the formation of a new profile based on class(es) from the previous step. The frame/box identifies the retained solution.
